# Foundation Model: A New Era for Plant Single-cell Genomics

**DOI:** 10.1093/gpbjnl/qzaf059

**Published:** 2025-06-25

**Authors:** Yuansong Zeng, Yuedong Yang

**Affiliations:** School of Big Data and Software Engineering, Chongqing University, Chongqing 400010, China; School of Computer Science and Engineering, Sun Yat-sen University, Guangzhou 510000, China; School of Computer Science and Engineering, Sun Yat-sen University, Guangzhou 510000, China

Single-cell RNA sequencing (scRNA-seq), introduced in 2009, has rapidly become a cornerstone of biological research, particularly in uncovering cellular heterogeneity, developmental trajectories, and gene regulatory networks. By enabling high-resolution analysis of gene expression at the single-cell level, scRNA-seq overcomes the limitations of traditional bulk RNA sequencing that averages gene expression across cell populations. This transformative technology has provided unprecedented insights into the cellular composition of complex tissues and organs, revealing rare cell types, transient states, and dynamic regulatory interactions that were previously obscured. Its applications span diverse fields, including developmental biology, immunology, and cancer research, where it has become an indispensable tool for dissecting cellular diversity, mapping lineage relationships, and identifying key drivers of disease progression [1]. scRNA-seq has become a core research method, driving breakthroughs in our understanding of cellular behavior and tissue organization.

With the continuous development of single-cell sequencing technologies, rapidly accumulating data have been leveraged to develop foundation models that learn the underlying patterns of cellular behavior. Notable examples include Geneformer [2], scGPT [3], scFoundation [4], GeneCompass [5], and CellFM [6], which were trained on increasingly large datasets ranging from 30 to 100 million cells, each contributing unique advancements to the field. These foundation models have not only advanced single-cell research but also paved the way for innovations in multi-omics integration, spatial transcriptomics, and precision medicine. As datasets continue to scale and model architectures evolve, the field is poised to unlock even deeper insights into cellular biology and disease mechanisms.

Despite the significant achievements of single-cell genomics in the animal model, the development of plant single-cell genomics has been relatively slow. The polyploidy, cell walls, and complex tissue-specific expression patterns of plant genomes present unique challenges for analyzing plant single-cell data. Existing single-cell computational models, primarily trained on animal datasets, have not been extensively tested on plant single-cell data. While these models have shown success in animal systems, their performance on plant data remains uncertain due to the lack of specialized training on plant-specific datasets. This highlights the need to develop single-cell analysis methods specifically tailored for plant data, which can address the unique challenges posed by plant cellular complexity, such as cell wall structures, polyploidy, and tissue-specific expression patterns as mentioned above.

To address these issues, Cao et al. introduced scPlantLLM [7], a transformer-based model specifically trained on plant single-cell data. scPlantLLM was pretrained using a combined optimization of masked language modeling and cell type annotation tasks to accurately capture the underlying patterns of gene expression in plant cells. One of the notable features of scPlantLLM is its excellent performance in zero-shot learning, maintaining high accuracy in cell type annotation and batch integration even on previously unseen plant species data.

Notably, scPlantLLM overcomes the issues found in traditional methods related to batch effect correction and cross-platform data integration. Compared to other deep learning models, the uniqueness of scPlantLLM lies in its ability to adapt to and effectively address the complexity of plant genomic data based on plant-specific biological features. scPlantLLM provides a new analytical approach for plant single-cell genomics, which helps researchers gain deeper insights into plant development, adaptability, and environmental response mechanisms.

With the continuous development of computational technologies and multi-omics data, single-cell genomics holds immense potential for advancing biological research. Inspired by single-cell foundational models, as well as artificial intelligence (AI)-driven approaches such as virtual cell construction [8] and cross-scale genome modeling tools like Evo2 [9], future single-cell analysis methods are expected to integrate multi-modal data, including transcriptomics, epigenomics, and cellular images. Techniques like cross-modal graph contrastive learning [10], which combine cellular images with transcriptomic data, could bridge structural and functional genomics, offering new insights into cellular behavior, development, and stress responses across diverse biological systems.

Looking ahead, the integration of spatial transcriptomics and single-cell epigenomics will further enhance our understanding of tissue organization and gene regulation. These advancements will not only enrich our knowledge of basic biological processes, but also drive innovations in plant-related fields such as precision agriculture, crop improvement, and stress resilience research.

## CRediT author statement

**Yuansong Zeng:** Writing − original draft. **Yuedong Yang:** Conceptualization, Writing − review & editing. Both authors have read and approved the final manuscript.

## Competing interests

Both authors have declared no competing interests.
